# Analysis of non-carcinogenic health risk assessment of elemental impurities in vitamin C supplements

**DOI:** 10.22038/IJBMS.2022.67487.14789

**Published:** 2023-02

**Authors:** Fadime Canbolat

**Affiliations:** 1 Department of Pharmacy Services, Vocational School of Health Services, Çanakkale Onsekiz Mart University, Çanakkale, Turkey

**Keywords:** Elemental ımpurıty, Hazard ındex, Hazard quotient, ICP-MS, Risk assessment, Supplements, Vitamin C

## Abstract

**Objective(s):**

Elemental impurity exposure that may occur in the use of supplements has the potential to pose a risk to human health. Vitamin C supplements are among the most commonly used supplements on a daily basis and in the long-term due to the pharmacological properties of vitamin C. In this study, we aimed to evaluate the non-carcinogenic health risk of elemental impurities that may cause contamination in orally administered vitamin C supplements.

**Materials and Methods:**

Ten elemental impurities (Cd, Pb, As, Hg, Co, V, Ni, Cr, Sb, and Sn) in 12 supplements were analyzed using ICP-MS. The estimated daily intake (EDI), hazard quotient (HQ), and hazard index (HI) values of elemental impurities were calculated for non-carcinogenic risk assessment. Cancer risk (CR) was additionally calculated for elemental impurities with carcinogenic properties detected in the samples.

**Results:**

Low levels of Cr and Hg were detected in some samples. While the HQ values of sample 1, sample 2, sample 8, and sample 9 for Hg were calculated as 0.054, 0.096, 0.064, and 0.086, respectively, the HQ values of sample 5, sample 10, and sample 11 for Cr were calculated as 0.011, 0.017, and 0.014, respectively. Since only Hg or only Cr was detected in samples with elemental impurity, the HI values in the samples are the same as the HQ values. Since the HQ and HI values calculated from the samples are not≥1, there is no elemental impurity at a level that will hazard human health through supplement use. Other carcinogenic elements were not detected in the samples except Cr. In sample 5, sample 10, and sample 11, the CR values for Cr were 1.767.10^-5^, 2.571.10^-5^, and 2.089.10^-5^, respectively. In probability simulation, while HQ and CR values of Cr did not exceed the allowable value, the HQ level for Hg in the 95% slice was higher than the allowable value.

**Conclusion:**

There is no risk to human health and there is no critical difference between the supplements considering the elemental pollutant content among the vitamin C supplements of different trademarks. However, in order to keep the Hg level, which has a potential risk capacity, at low limits, it is recommended that the necessary risk-reducing measures be taken by the authorities and further studies be carried out.

## Introduction

The frequency and popularity of nutritional supplements are increasing daily worldwide. Studies have shown that half of the adults are using nutritional supplements. According to the European Food Safety Authority (EFSA) 2022 technical report, Finland, Hungary, and the Netherlands are among the countries that consume most food supplements in Europe. In Finland, 51% of the children, 35.9% of the young people, 59.3% of the adults, and 75.4% of the elderly use food supplements. This rate is gradually decreasing in other European countries (1) The reasons for this widespread use include finding food supplements safe and effective, sales strategies, advertisements, easy accessibility via the internet without a prescription, and the frequency of use depending on the initiative of the person (2, 3). It has been stated in studies that the nutritional supplements with the highest amount of use are multivitamins with or without mineral content (3). In long-term use, the vitamin supplement with the highest daily consumption is vitamin C (4). Vitamin C is one of the most sold nutrients in the United States (US) vitamin and mineral market, and healthy people are predominantly the highest-ranking buyers (4). Vitamin C is an essential micronutrient required for various biochemical and physiological processes in the body (5). Vitamin C, which is found in many fruits and vegetables, cannot be synthesized in the human body, so it must be taken from the diet. Both the reduced form L-ascorbic acid and the oxidized form L-dehydroascorbic acid (DHA) preserve vitamin C activity (6, 7). Since vitamin C plays an important role in metabolic functions, it is known that adequate consumption of vitamin C is effective in maintaining normal body functions (7-9). Vitamin C is known to play a role in important enzymatic reactions in the human body (e.g., collagen hydroxylation, carnitine biosynthesis, tyrosine metabolism). With its antioxidant properties, it can protect the body against oxidative stress. Regular use of vitamin C is known to strengthen the immune system and reduce the risk of cancer, cataracts, heart disease, and stroke (10). In order to derive the expected benefits from vitamin C, the vitamin C level must be at a steady-state concentration in the plasma. However, vitamin C is a water-soluble vitamin that can be excreted with urine without being stored in the body. Regular intake is needed to keep the concentration in the body at the desired level. Vitamin C supplements are among the most commonly used supplements on a daily basis and in the long-term due to the pharmacological properties of vitamin C. In a 1999 study, the World Health Organization (WHO, 2005) and EFSA (2006) reports indicate that 1000 mg/day of vitamin C use provides steady-state concentrations of vitamin C in the plasma. The recommended daily consumption of vitamin C is 1000 mg (10-12). Therefore, most vitamin C supplements contain 1000 mg of vitamin C. However, while trying to meet the lack of vitamins in our body with supplements, unwanted substances can also enter our bodies due to their additional contents. While the presence and amount of many substances in the supplement content are reported on the labels, there may also be elemental impurities with toxic properties that may be mixed unintentionally during production.

Vitamin C supplements contain acidity regulators, humectants, anti-caking agents, sweeteners, and colorants, as well as L-ascorbic acid. Elemental residues that are added during the production process and storage of drugs or nutritional supplements come unintentionally with the raw material or are added deliberately for therapeutic effectiveness can be included in the supplements (13). Therefore, the content of these products may differ from one manufacturer to another. It can be contaminated by heavy metals in the production process and affect the quality of the product. Control of elemental impurities in pharmaceuticals and nutritional supplements is very important in the marketing of pharmaceutical products. For this reason, the European Medicines Agency (EMA), United States Pharmacopeia (USP), and European Pharmacopoeia (Eur. Ph.) established joint working groups called the International Conference on Harmonization (ICH) and developed guidelines for the risk assessment of elemental residues in pharmaceuticals (14, 15). The toxicity limits of 24 elemental impurities are specified in USP <232> and ICH -Q3D on elemental impurities guidelines to identify elemental impurities in pharmaceuticals and nutritional supplements and to carry out a risk assessment. It is important to determine the permitted daily exposure (PDE) for elemental impurities (16). Permitted daily exposure limits vary depending on the element and the route of administration of the pharmaceuticals. The condition of exposure to elemental impurities (intentional or unintentional, oral, parenteral, and inhalation) may affect the need for risk assessment.

Element classifications are included in the guidelines according to the necessity of risk assessment (17, 18). The risk assessment classification according to the toxic effects of elemental impurities is divided into three classes in the USP <232> and EMA ICH guideline Q3D (18-20). In Class 1, elements are human toxicants across all administration routes. These require special consideration during risk assessment due to their high toxicity and the potential for them to be present in finished dosage form through contributions of naturally derived materials. In Class 2, human toxicants are to a greater or lesser extent based on the route of administration. In class 3, relatively low toxicity by the oral route of administration exists but requires consideration in the risk assessment for other routes of administration (18-20). According to the USP (2017) and EMA (2019), elemental impurity guidelines to identify elemental impurities in pharmaceuticals and nutritional supplements, risk assessment of the elements in Class 1 (cadmium (Cd), lead (Pb), arsenic (As), and mercury (Hg)) and Class 2A (cobalt (Co), vanadium (V), and nickel (Ni)) is required under all circumstances. The risk assessment of Class 2B elements is required if they are intentionally added to pharmaceuticals. Antimony (Sb), tin (Sn), and chromium (Cr) are among the elements in Class 3 (18, 20). The risk assessment of the elements in Class 1 and Class 2A for orally administered pharmaceuticals is specified in the guide ((18-20). USP <233> describes the validation procedure of analytical techniques used to analyze elemental impurities specified in USP <232> (20, 21). The analytical method procedures of inductively coupled plasma‒optical emission spectrometry (ICP-OES) and inductively coupled plasma‒mass spectrometry (ICP‒MS) instruments used in elemental impurity analysis are described in the USP <233> (21, 22). Methods of sample preparation of pharmaceuticals or nutritional supplements by direct dissolution in aqueous or organic solvents, wet digestion, or microwave-assisted analysis are also included in the USP <233> (21, 22). In elemental impurity analysis, the ICP‒MS method is a widely used technique in the literature for the quantification of trace elements due to its high sensitivity.

Elemental impurity exposure that may occur in the use of food supplements has the potential to pose a risk to human health. Therefore, the PDE limits of these elements are given in the USP guideline (18). Toxic symptoms may occur when these limits are exceeded. The risk assessment accounts for the assessment of the hazard in the event of exposure to a chemical. The risk to human health is quantified in terms of non-carcinogenic and carcinogenic risk factors. In the characterization of non-carcinogenic risk, the reference dose factor (RfD) of the United States Environmental Protection Agency (USEPA) is used, and these organization-approved values are listed in the Integrated Risk Information System (IRIS) (17, 23). The hazard quotient (HQ) for hazard determination in elemental impurity exposure is obtained by dividing the estimated daily intake by RfD. In cases where HQ ≥ 1, the health risk is high. The hazard index (HI) of the cumulative risk associated with the elemental impurities analyzed is calculated using the sum of the HQs. The hazard index value ≥ 1 indicates that exposure to elemental impurities is risky for human health (23, 24).

The body can be exposed to elemental impurities that can be mixed into the supplement content with daily and long-term vitamin C supplement consumption. The fact that elemental impurities can be taken into the body every day with the use of vitamin C supplements increases the risk of elemental toxicity. Therefore, in our study, the focused matrix and vitamin C supplement were selected to determine the risk of elemental impurity in drug and drug-like substances. In this study, we aimed to evaluate the non-carcinogenic risk of 10 elemental impurities (Cd, Pb, As, Hg, Co, V, Ni, Cr, Sb, and Sn) that may cause contamination in orally administered vitamin C supplements.

## Materials and Methods

***Reagents and Materials***

Common 10 ppm multielement ICP‒MS 71 A standard (Cd, Pb, As, Co, V, and Ni) in HNO_3_, 10 ppm multielement ICP‒MS 71 B (Sb and Sn) in nitric acid (HNO_3_) and 10 µg/ml Hg in hydrochloric acid (HCl) were purchased from Inorganic Ventures (Virginia, USA). Ten µg/ml potassium dichromate (K_2_Cr_2_O_7_) in deionized water was purchased from Chemlab (Belgium). Nitric acid (HNO_3_; 65%) and hydrogen peroxide (H_2_O_2_; ≥30%) for trace analysis were purchased from Sigma Aldrich (USA). Ultrapure water was used for sample dilutions. Ultrapure water (18 M^-cm^) was produced through a Milli-Q water purification system (Millipore, Bedford, Massachusetts, USA).

***Preparation of standard solution***

Ten elemental impurities (Cd, Pb, As, Hg, Co, V, Ni, Cr, Sb, and Sn), including Class 1 and Class 2A elements, which are recommended to be evaluated in oral use in USP <232>, should be analyzed using the ICP‒MS microwave sample preparation method. Standard solutions for the determined elements were diluted with ultrapure water by appropriate dilution of multi-element ICP standard solutions (ICP‒MS 71 A; ICP‒MS 71 B), K_2_Cr_2_O_7_, and Hg. While Cd, Pb, As, Co, V, and Ni standard concentrations ranged from 0.1 to 1000 ppb, Sb and Sn ranged from 0.01 to 500 ppb. While the concentration of Cr ranged from 0.01 to 50 ppb, the concentration of Hg ranged from 0.005 to 2.0 ppb to reduce the potential for memory effects and element carryover.

***Sample collection***

Twelve different vitamin C supplements were purchased from the online market. The supplements were in the form of effervescent tablets containing 1000 mg of vitamin C (Table 1). As the recommended daily vitamin C consumption in the literature is 1000 mg (10, 12), each effervescent tablet purchased in the study contains 1000 mg of vitamin C. Also, the recommended daily intake (IR) of the purchased products is reported on company labels as one effervescent tablet (effervescent tablet weight about 4.5 g) per day per capita.

***Sample preparation***

Closed vessel microwave digestion is recognized as the most universal sample preparation method for materials for subsequent elemental analysis by ICP‒MS. All samples were brought into solution via a microwave digestion procedure using an UltraWAVE closed vessel microwave digestion system (Milestone, Sorisole, Italy). Since the supplements are water soluble, they were prepared for analysis via direct dissolution in dilute HNO_3_ and H_2_O_2_ by modifying Rudovica *et al.* (2014), Barin *et al.* (2016), Su *et al.* (2020) and Pinheiro *et al.* (2020) study methods (19, 22, 24, 25). Each tablet was ground; then each sample was weighed 0.4 g into 15 ml disposable glass vials. Then, 8 ml of HNO_3_ (65%, v/v) and 2 ml of H_2_O_2_ (30%, v/v) were added to each vial (c: 40 mg/ml). The sample vials were transferred into the microwave digestion system, which was then closed and pressurized with nitrogen at 40 bar, and the temperature program shown in Table 2 was launched. Digested samples were transferred to polypropylene vials, and digested samples were diluted 1/4 with ultrapure water (working solution 1 (WS1); c_ws1_10 mg/ml). Then, each WS1 was diluted 1/100 with ultrapure water (working solution 2 (WS2); c_ws2_: 100 µg/ml). Next, each WS2 was diluted 1/5 with ultrapure water (working solution 3 (WS3); c_WS3_: 20 µg/ml). Finally, each WS3 (20000-fold diluted) was transferred to 15 ml polypropylene autosampler vials and given to the ICP‒MS system. Each vitamin C supplement was analyzed as three repetitions.

***Instrumentation***

All measurements were performed using a Thermo Scientific™ iCAP™ RQ ICP‒MS. The instrument was operated using the Thermo Scientific Qtegra™ Intelligent Scientific Data Solution (ISDS™) Software and was calibrated daily using a mixed standard solution.

The microwave digestion program was set as follows: microwave power of 1800 Wt, temperature of 200 ^°^C, and time of 30 min. The operation parameters of ICP‒MS were set as follows: plasma power (RF) was 1600 W, Helium was makeup gas, and plasma gas (Ar) flow was 14.8 Liter/min. The nebulizer pump was 40 rpm, the sampling depth was 15.00 mm, the scanning speed was 3000 amu/second, and the mass range was 2-260 amu. The limit of detection (LOD) value for Cd, Pb, As, Co, and Ni in ICP‒MS analyses of calibration standards prepared at different concentrations was 0.01 ppb. The LOD value for V, Sb, and Sn is 0.002 ppb. The LOD value for Cr and Hg is 0.001 ppb. The calibration range of the method was 0.1-1000 ppb for Cd, Pb, As, Co, V, and Ni. The calibration range for Sb and Sn was 0.01-500 ppb. The calibration range was 0.01-50 ppb for Cr and 0.005–2 ppb for Hg. The level of elemental impurities in samples was measured at the ppb level by ICP‒MS.

***Risk assessment for human health***

The estimated daily intake (EDI), HQ, and HI values of elemental impurities were calculated for the non-carcinogenic risk assessment of elemental impurities in vitamin C supplements. Cancer risk (CR) was additionally calculated for elemental impurities with carcinogenic properties detected in the samples.

The levels of elements measured at the ppb level by ICP‒MS to find EDI, HQ, and HI values in samples were converted to µg/g using equation 1. For equation 1, the study of Amariei *et al.* 2021 was taken into account (18). In equation 1, the weight of the effervescent tablet used in the sample preparation analysis and the final diluted volume of the sample given for analysis were included in the calculation (Equation 1) (26). The conversion coefficient (CF) is used for unit conversion in the equation.

C _µg/g_ = (a. V/m)*CF_1 _*(Equation 1)*

C= Amount of elemental impurity; µg/g

a= Elemental impurity level read in ICP‒MS; ppb

V= Total diluted volume of dissolving effervescent tablet in sample preparation analysis; ml

m= Weight of dissolving effervescent tablet in sample preparation analysis; (g)

CF_1_= Conversion coefficient (from ng to µg; 10^-3^)

***Calculation of the non-carcinogenic health risk for adult***

Non-carcinogenic risk estimation analysis of elemental impurities was calculated according to HQ. To calculate the HQ, the EDI value of each element was determined initially. The EDI value was calculated according to equation 2, taking into account the body weight of an average adult person of 70 kg (23, 27, 28). The conversion coefficient (CF) is used for unit conversion in equation 2.

EDI (mg/kg/day)=(EF x ED x IR x C)* CF_2_/(BW xAT)*(Equation 2)*

EF: Exposure frequency (365 days/year) (28)

ED: Exposure duration (30 years for adults) (28)

IR: Recommended daily intake of effervescent dose. Because the recommended daily dose in the supplement boxes was labeled as 1 tablet (4.5 g) per day per capita, 4.5 g was used as the IR value.

C: Amount of elemental impurity in the supplement; µg/g

BW: Body weight; it was considered 70 kg for adults (27, 29) 

AT: Average time (days), which is ED×EF (28, 30)

CF_2_: Conversion coefficient (from µg to mg; 10^-3^)

The hazard quotient (HQ) value was calculated using the calculated EDI value and the oral reference dose (RfD) value of each elemental impurity (Equation 3) (31). The USEPA has slightly modified the Acceptable Daily Intake (ADI) approach and calculates a reference dose (RfD) as the acceptable safety level for chronic noncarcinogenic and developmental effects. It is determined by applying safety factors (to account for the uncertainty in the data) to the highest dose in human or animal studies, which has been demonstrated not to cause toxicity (no-observed-adverse-effect level (NOAEL)). In determining the RfDs, the NOAEL is divided by safety factors (uncertainty factors) to provide a margin of safety for allowable human exposure (17) If the hazard coefficient is ≥ 1, it indicates that the amount of the element exposed is a risk to human health (24). The oral RfD (mg /kg/day) and oral PDE (µg/day) limits of the 10 elements are given in Table 2.

HQ = EDI/RfD *(Equation 3)*

HQ: The hazard quotient 

EDI: Estimated daily intake dose; mg/kg/day

RfD: Oral reference dose; mg/kg/day.

The hazard index (HI) of the cumulative risk associated with the analyzed metals was calculated using the sum of the HQs for Cd, Pb, As, Hg, Co, V, Ni, Cr, Sb, and Sn for each sample (Equation 4) (24, 31).

HI=HQ (Cd)+HQ (Pb)+HQ (As)+HQ (Hg)+HQ (Co)+HQ (V)+HQ (Ni)+HQ (Cr)+HQ (Sb)+HQ (Sn) *(Equation 4)*

A hazard index (HI) value equal to or less than 1 indicates that exposure to these metals is not expected to cause adverse non-carcinogenic effects (24, 31). However, an HI value greater than 1, according to the EPA, cannot be suggested as a statistical probability that adverse health effects will occur.

***Calculation of the carcinogenic health risk for adult***

Daily exposure to some potential carcinogens will increase the possibility of cancer development in humans. The cancer risk (CR) due to life-long exposure to toxic elements can be calculated using the cancer slope factor (CSF) and the following equation 5 (28, 30). 

CR= EDI x CSF *(Equation 5)*

CR: cancer risk

EDI: Estimated daily intake dose; mg/kg/day

CSF: The cancer slope factor; (mg/kg/day) ^−1^

The cancer slope factor (CSF) values for carcinogenic metals, including Cd, Cr, Pb, Ni, and As, are 0.38, 0.5, 0.0085, 1.7, and 1.5 (mg/kg/day)^−1^, respectively (40-42). According to the USEPA, a CR value between 1×10^-6^ and 1×10^-4^ is acceptable for humans. However, the CR value should not exceed 1×10^-4^ (43).

***Probability simulation analysis***

The simulated probability analysis was performed by modifying the Pirsaheb** *et al.* 2021 study (28). After calculating the point estimates of HQ and CR using equations (3) and (5), the simulation technique with 10,000 maximum cases was run in SPSS to estimate the HQ and CR considering the distribution of independent variables (IR, BW, and C).

A one-unit change in independent variables may have the potential to affect the results in equations 3 and 5 in different directions. Therefore, to determine the effect of uncertainty and sensitivity arising from these differences, the independent variable levels in the literature were taken into account while performing the probability analysis. Schlueter *et al.* (2010) reported that the daily tolerable dose of vitamin C supplements could increase up to 2000 mg (44). Therefore, in our probability analysis, the IR value was increased to vitamin C supplement weights equivalent to 2000 mg vitamin C content. In a 2020 study, risk assessment of elemental impurity levels in food supplements was included. In their study, risk calculations were made by considering the ranges of 50-90 kg as adult weight (23). Similarly, in Pirsaheb *et al.* 2021 study, the adult BW range used in the Monte Carlo analysis for the risk calculation of elemental impurity in foods was taken as 60-80 kg. In our study, the BW range considered in our probability analysis is 50-90 kg to comply with the literature (23, 28). In addition, elemental impurity levels detected in drug and food supplements in the literature have been taken into account in the probability calculation (14, 27, 34, 45).

Then, by comparing the uncertainty upper bound (the 95th percentile; P95) and the uncertainty lower bound (5th percentile; P5) with the permitted values (HQ<1 and CR<10^-4^), a final decision on the health risk of each of the toxic metals in samples was presented. In the present study, the estimated values for HQ and CR were determined for the upper bound (P95) and the lower bound (P5).

## Results

***Result of ICP-MS analysis***

**Figure 1:**
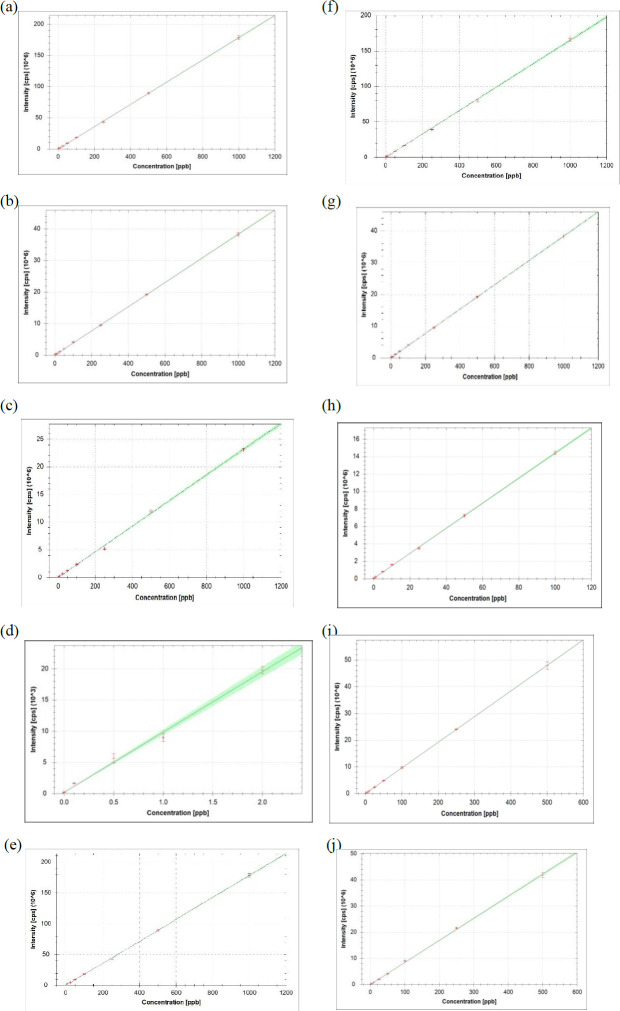
Calibration curve of elemental impurities

Ten elemental impurities (Cd, Pb, As, Hg, Co, V, Ni, Cr, Sb, and Sn), also including Class 1 and Class 2A elements in the USP <232> guideline, were analyzed in the ICP‒MS device using the microwave sample preparation method. Samples were prepared, analyzed, and evaluated according to criteria set out in the USP <232> and USP <233> guidelines. In our study, the limit of detection (LOD) value for Cd, Pb, As, Co, and Ni in ICP‒MS analyses of calibration standards prepared at different concentrations was 0.01 ppb. The LOD value for V, Sb, and Sn is 0.002 ppb. The LOD value for Cr and Hg is 0.001 ppb. The calibration range of the method was 0.1-1000 ppb for Cd, Pb, As, Co, V, and Ni. The calibration range for Sb and Sn was 0.01-500 ppb. The calibration range was 0.01-50 ppb for Cr and 0.005-2 ppb for Hg. The regression value of the calibration curve for all elements was R^2^≥0.9939 (Figure 1). Table 3 contains the ICP‒MS calibration curve information and LOD values determined by the calibration standards of the 10 elements.

Analysis of 12 vitamin C effervescent tablets showed that Cd, Pb, As, Co, V, Ni, Sb, and Sn were not detected in any of the samples. Hg was detected in four of the twelve samples. The elemental impurity of Hg was detected within the calibration range in samples 1, 2, 8, and 9. Cr was detected in three of twelve samples. The elemental impurity of Cr was detected within the calibration range in samples 5, 10, and 11. More than one elemental impurity was not detected in any sample. The elemental impurity level measured at the ppb level by ICP‒MS specified in Table 4 was converted to the “calculated amount of elemental impurity; µg/g” by using equation 1.

***Result of the health risk assessment***

The amount of elemental impurities calculated in Table 4, the level of elemental impurity at ppb, the weight of the effervescent tablet used in the analysis, and the final diluted volumes of the samples were obtained by placing them in equation 1. Thus, The amount of elemental impurity calculated in Table 4 was converted to µg/g by placing the effervescent tablet weight used in the analysis, the level of elemental impurity (ppb level), and the final diluted volumes of the samples given for analysis into equation 1. In Table 4, the EDI, HQ, HI, and CR values ​​obtained using equations 2-5 are shown.

The formula in equation 2 was used to determine the EDI (mg/kg/day) of each element in the analyzed supplement samples. The “calculated amount of elemental impurity; µg/g” in Table 4 was used for the amount of elemental impurity in the formula. Because the recommended daily dose in the supplement boxes was labeled as 1 tablet (4.5 g) per day per capita, 4.5 g was used as the IR value. Therefore IR was given as “4.5 g” in Table 4. The calculation in equation 2 was made considering an adult with an average weight of 70 kg to calculate the EDI (mg/kg/day) for elemental impurities (29, 46). The results are given in Table 4. To determine the HQ value, the calculation in equation 3 was made by using the oral RfD value of each element in Table 2 and the EDI values calculated in Table 4. The results are given in Table 4. When Table 4 is examined, the mean Hg impurity levels of sample 1, sample 2, sample 8, and sample 9 measured by ICP‒MS are 0.005±0.002, 0.009±0.001, 0.006±0.003, and 0.008±0.002 ppb, respectively. The mean Hg elemental impurity levels of sample 1, sample 2, sample 8, and sample 9 converted from the ppb level to the µg/g level using Equation 1 were 0.25±0.015, 0.45±0.012, 0.30±0.017, and 0.40±0.0130 µg/g, respectively. The calculated EDI values of sample 1, sample 2, sample 8, and sample 9 for Hg were 1.6.10^-5^, 2.9.10^-5^, 1.9.10^-5^, and 2.6.10^-5^ mg/kg/day, respectively. The calculated HQ values of sample 1, sample 2, sample 8, and sample 9 for Hg were calculated as 0.054, 0.096, 0.064, and 0.086, respectively. The calculated HQ values of the Hg elemental impurity in these samples were <1.

When Table 4 is examined, the mean elemental impurity levels of Cr, measured with ICP‒MS in sample 5, sample 10, and sample 11 were 0.011±0.004, 0.016±0.007, and 0.013±0.003 ppb, respectively. The mean Cr elemental impurity levels of sample 5, sample 10, and sample 11 converted from the ppb level to the µg/g level using Equation 1 were 0.55±0.002, 0.80±0.005, and 0.65±0.003 µg/g, respectively. The calculated EDI values of sample 5, sample 10, and sample 11 for Cr were 3.5.10^-5^, 5.1.10^-5^, and 4.2.10^-5^ mg/kg/day, respectively. The calculated HQ values of sample 5, sample 10, and sample 11 for Cr were 0.011, 0.017, and 0.014, respectively. The calculated HQ values of the Cr elemental impurity in these samples were <1. Since the HQ values calculated from the samples are not ≥ 1, there is no elemental impurity at a level that will hazard human health during supplement use.

When Table 4 is examined, none of the 10 element impurities were detected in sample 3, sample 4, sample 6, sample 7, and sample 12. Since only Hg or only Cr was detected in samples with elemental impurity, the HI values in the samples are the same as the HQ values (Table 3). Since the HI value is not ≥ 1, elemental impurity at a level that can hazard human health during supplement use is not present.

In the elemental impurity analysis of the samples, only the Cr element, which is one of the carcinogenic elements, was detected in sample 5, sample 10, and sample 11. Other carcinogenic elements were not detected in the samples. In sample 5, sample 10, and sample 11, the CR values calculated by using equation 5 for Cr were 1.767.10 ^-5^, 2.571.10^-5^, and 2.089.10^-5^, respectively (Table 4).

***Result of probability simulation analysis*** *.*

**Figure 2:**
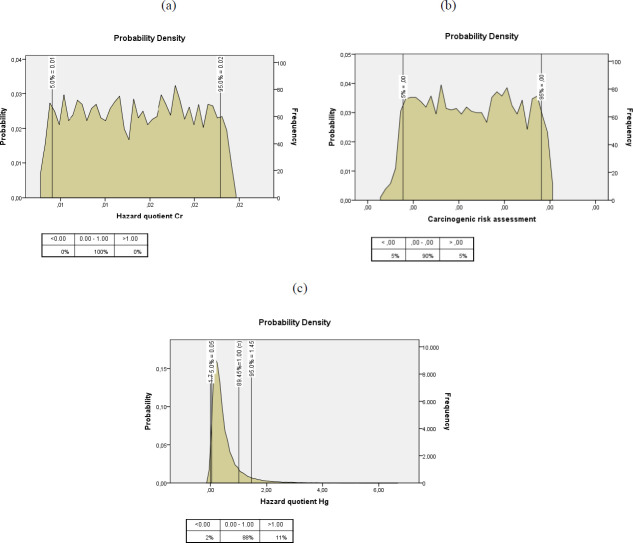
Result of probability simulation analysis for Cr and Hg elemental impurities

In the probability analysis performed using the simulation method for Cr and Hg, which were identified as elemental impurities in the samples, the effects of the change in BW, IR, and C values (independent variables) on HQ and CR values were determined. HQ and CR probability calculations were performed for the Cr elemental impurity with carcinogenic properties, while only HQ evaluation was performed for Hg. In Cr elemental impurity, the correlation between BW and HQ was found to be significant (*P*<0.05). The correlation between the BW and CR values of the Cr element was also found to be significant (*P*<0.05). According to probability simulation results shown in Figure 2, the uncertainty upper bound (P95) corresponding to HQ and CR value for Cr elemental impurity was lower than the allowable value (HQ <1; CR <10-4). In Figure 2, the P80 corresponding to HQ-Hg was equal to the allowable value, and P95 was higher than the allowable value.

## Discussion

The presence of the element in food and vitamin supplements can be found intentionally or undesirably as a result of contamination as in pharmaceuticals. Causes of elemental impurities being mixed into supplements as a result of contamination include metal catalysts, raw material pollutants, metal reagents used during synthesis, and problems experienced during production, transportation, storage, and packaging (16). It is important for health to follow up on the elemental impurities that may be found as a result of contamination in oral supplements. As, Cd, Hg, and Pb elements in the Class 1 group are toxic elements whose use in the production of pharmaceuticals should be limited or prohibited. Co, Ni, and V elements in the Class 2A group are elements that are likely to occur during the production phase of pharmaceuticals. Although Cr, Sb, and Sn elements in the Class 3 group cause mild toxicity when taken orally, they are likely to be found in pharmaceuticals (19). Some essential elements can be sold in the form of mineral-vitamin supplements, especially by deliberately adding them to vitamin supplements. However, it is important to prefer vitamins that do not contain mineral supplements for analysis to detect elemental impurities that may occur as a result of contamination in vitamin supplements. The fact that elemental impurities can be taken into the body every day with the use of vitamin C increases the risk of elemental toxicity. Therefore, in our study, the focused matrix and vitamin C supplement were selected to determine the risk of elemental impurity in drug and drug-like substances. In our study, 12 vitamin C supplements, the contents of which are specified in Table 1 and which do not contain minerals, were analyzed.

Since elemental impurities pose a risk to human health, monitoring elemental impurities in pharmaceuticals is vital. Analyses performed on high-sensitivity ICP‒MS devices using the closed system microwave sample preparation method are included among the most preferred analysis methods for elemental impurity analysis in USP, EMA guidelines, and the literature (20, 21). Barin *et al.* included elemental impurity analyses of pharmaceuticals in their 2016 review. In this review, it was reported that tablets were dissolved in HNO_3_:H_2_O_2_ solvents for the analysis of As, Cd, Co, Cr, Ni, and Pb elements in antibiotic tablets. In the same review, it was reported that a closed-system microwave sample preparation procedure was used by dissolving a different drug group in HNO_3_:H_2_O_2_ solvents (19). In the study of Rudovica *et al.* in 2016, solid pharmaceuticals were dissolved in 6 ml of 65% HNO_3_ and 2 ml of 30% H_2_O_2_ solvents for elemental impurity analysis of pharmaceuticals. Then, the samples prepared with the closed system microwave sample preparation procedure were analyzed by ICP‒MS (22). In the 2020 study by Su *et al*., analysis was carried out in a closed microwave system using HNO_3 _and H_2_O_2_ solvents for the determination of Cr, As, Cd, and Pb in solid food supplements. Samples were analyzed by ICP‒MS (24). In our study, the HNO_3_ and H_2_O_2_ solvents recommended in the literature and guidelines were preferred in the sample preparation procedure to detect elemental impurities in vitamin C supplements. In our study, the supplements were analyzed in the ICP‒MS using the closed-system microwave sample preparation procedure. In our study, the use of the sample preparation procedure, which has been proven in the literature and recommended for analysis, increases the reliability of our results.

When the studies using ICP‒MS for elemental impurity analyses are examined, the LOD values for Cd, Cr, Ni, and Pb in the ICP‒MS method in one study are 0.02, 0.17, 0.06, and 0.03 ppb, respectively (47). In a study by Gu *et al.* in 2021, elemental impurity analysis in oral pharmaceuticals was performed using ICP‒MS. In Gu *et al*. study, the LOD values of the analyzed elements Cd, Pb, As, Hg, Co, V, Ni, Cr, Sb, and Sn were 0.003, 0.002, 0.03, 0.007, 0.002, 0.03, 0.01, 0.09, 0.07, and 0.03 ppb, respectively (34). The LOD values of Cd, Pb, As, Hg, Co, V, Ni, Cr, Sb, and Sn elements in our study were 0.01, 0.01, 0.01, 0.001, 0.01, 0.002, 0.01, 0.001, 0.002, and 0.002 ppb, respectively. The lowest calibration curve R^2 ^value for the 10 elemental impurities analyzed in our study was 0.9939 (Table 3). When the method sensitivity of the ICP‒MS methods used for elemental analysis in the literature is compared with our study, it is seen that the ICP‒MS sensitivity and method accuracy in our study are similar to those in the literature.

A 2022 study analyzed As, Cr, Cd, and Pb elements that can be mixed in fresh water in China with the ICP-MS device (43). At the end of the study, it was determined that the Cd element level in organisms living in an aquatic environment was above acceptable limits. The importance of monitoring the toxic elements that can be mixed with food for food safety and human health was emphasized in the study (43). In a 2020 study, the effect of As, Cd, Hg, and Pb concentrations in protein powders on human health was examined by determining the HI value (48). In the study, no sample had a HI value>1. In the study, it is reported that elemental impurity levels that may be mixed with the daily recommended amount of consumed protein powders are not harmful to health (48). In our study, elemental impurity analysis was performed on 12 vitamin C effervescent tablet supplements. Cd, Pb, As, Co, V, Ni, Sb, and Sn were not detected in any of the samples (< LOD). Hg was detected in four of the twelve samples. The elemental impurity of Hg was detected within the calibration range in samples 1, 2, 8, and 9. Cr was detected in three of twelve samples. The elemental impurity of Cr was detected within the calibration range in samples 5, 10, and 11. More than one elemental impurity was not detected in any sample. 

In the study of Amariei *et al.* (2017), the risk assessment of elemental impurities involved in food supplements from different sources was carried out. At the end of the analysis, it was reported that the risk potential on health was negligible since the HI values of the elements Cr, As, Hg, and Pb were found to be low (26). In our study, negligible elemental impurities were detected. The data in our study are similar to those in the literature.

Cadmium (Cd) is generally found at low levels in nature and is obtained as a byproduct (waste product) of zinc, lead, and copper mining (37). USEPA reported the RfD value of Cd as 0.5 µg/kg/day and the PDE value of Cd as 5 µg/day. (33). Abnormalities in kidney, liver, skeletal and reproductive functions have been reported at exposures above this cutoff value (49). In our study, Cd levels in vitamin C samples could not be detected because Cd was below the detection limit (<0.01; LOD). It has been determined that there is no risk potential arising from Cd in the supplements included in our study.

Lead (Pb) poisoning is more common in toxic metal poisoning caused by the consumption of herbal medicines compared to other metals. It has been reported that Pb is detected in the blood of patients presenting with abdominal pain and vomiting as a result of herbal drug use (50). It is known that Pb can cause memory loss, intellectual disabilities, abnormal neurotransmitter functions, cardiovascular diseases, and kidney and liver damage (37). The oral RfD value of Pb has been reported to be 3.5 µg/kg/day and the PDE value of Pb is 5 µg/day (23, 29, 42). In our study, Pb levels in vitamin C samples could not be detected because they were below the detection limit (< 0.01; LOD). It has been determined that there is no risk potential arising from Pb in the supplements included in the analysis. 

In the 2020 study by Ćwieląg-Drabe *et al*., elemental impurities in 41 dietary supplements were analyzed. Cd and Pb contamination was found in 68.3% of 41 dietary supplements. While some of the Cd and Pb contaminations were not at a level to affect human health, the HQ value was greater than one in some of them (20). In our study, Cd and Pb were not detected in any of the samples. The samples do not have the potential to be dangerous in terms of Cd and Pb toxicity (27). In a study by Amariei *et al.* in 2017, elemental impurity analysis was performed on food supplements of animal, vegetable, and mineral origin. The HQ value of Cd in these supplements was 11.31 in those of animal origin, 2.81 in those of mineral origin, and 5.89 in those of vegetable origin. Since HQ values are greater than one for Cd, these supplements carry a potential health risk. The calculated HQ value of other elements (Cr, As, Hg, and Pb) analyzed in the same products was determined to be below the risk limit (HQ<1). It was reported that the consumption of food supplements in terms of Cr, As, Hg, and Pb was not dangerous (26).

Arsenic (As) is one of the toxic elements that can enter organisms through water and food. The reported oral RfD value for As is 0.3 µg/kg/day. It has been reported that dermatitis, decreased neuron conduction, and liver carcinoma may develop as a result of exposures above this cutoff value (37). The reported PDE for As is 15 µg/day. In our study, As levels in vitamin C samples could not be detected because they were below the detection limit (<0.01; LOD). It has been determined that there is no risk potential arising from As in the supplements included in the analysis. 

Cobalt (Co) is one of the essential elements necessary for life. It is the main component of vitamin B12, which provides blood formation. Environmental exposure to Co is also common. The oral RfD value of Co is reported as 0.3 µg/kg/day, and the PDE value of Co is reported as 50 µg/day (36). It has been reported that exposure to Co may occur in alveoli, bronchial tumors, acute inflammation, alveolar epithelial hyperplasia, bronchial necrosis, and lung cancer. In our study, Co levels in vitamin C samples could not be determined because they were below the detection limit (<0.01; LOD). It has been determined that there is no risk potential due to Co in the supplements included in the analysis. Similarly, elemental impurity analysis was performed on medicinal plants in the Tschinkel *et al.* 2020 study. It has been reported that the Cd, Co, Cr, and Pb elemental impurity levels in the samples were below the detection limits (51).

In general, the toxicity of vanadium compounds is low. Food is the most important source of vanadium for humans. The oral RfD value of V has been reported to be 5 µg/kg/day and the PDE of V is 100 µg/day. In our study, the level of V in vitamin C samples could not be detected because it was below the detection limit (<0.002; LOD). It has been determined that there is no risk potential arising from V in the supplements included in the analysis

Nickel (Ni) is found in the Earth’s crust in trace amounts and is known to play a role as a cofactor in organisms. The oral RfD value of Ni as 20 µg/kg/day, and the PDE value of Ni as 200 µg/day were reported by the USEPA (37). Circulatory disorders and carcinogenic effects are reported at exposures above this value. In our study, Ni levels in vitamin C samples could not be detected because they were below the detection limit (<0.01; LOD). It has been determined that there is no risk potential due to Ni among the supplements included in the analysis. Similarly, in a study by Rudovica *et al.* in 2014, elemental impurities of Ni and Co in five pharmaceutical products were analyzed with an ICP‒MS instrument. HNO_3_ and H_2_O_2_ were used in the sample preparation procedure. Elemental impurity levels were found to be below the lowest quantifiable level of the method (<LOQ) (22).

Antimony (Sb) exposure can negatively affect the immune system, nervous system, respiratory and digestive systems, and other systems of the body. The oral RfD value of Sb was reported to be 0.4 µg/kg/day and the PDE value of Sb is 1200 µg/day (33). In our study, the level of Sb in vitamin C samples could not be determined since it was below the detection limit (< 0.002; LOD). It has been determined that there is no risk potential due to Sb present in the supplements included in the analysis.

Tin (Sn) can pass to the organism with the consumption of canned food, toothpaste, or seafood. The oral RfD value of Sn was reported as 600 µg/kg/day and the PDE value of Sn is 6000 µg/day (33). High levels of Sn have been reported to cause renal and hepatic dysfunction, gastrointestinal irritation, diarrhea, nausea, vomiting, and anemia. Sn levels in vitamin C samples could not be determined in our study because they were below the detection limit (<0.002; LOD). It has been determined that there is no risk potential due to Sn in the supplements included in the analysis.

Mercury (Hg) is widely present in nature in different forms. It exists mainly in the form of metal and inorganic Hg in nature. In its metallic form, Hg is liquid and does not make a compound with other elements. Inorganic Hg occurs in nature as mercury salts. Mercury which is in organic form is produced by microorganisms as a result of biological processes. Inorganic Hg is mostly used in health care, antiseptics, and creams. All forms of Hg can cause toxic findings. The nervous system, kidneys, respiratory system, immune system, gums, and skin are affected by Hg poisoning. The oral RfD value of Hg was reported to be 0.3 µg/kg/day and the PDE value of Hg is 30 µg/day (33). In our study, Hg elemental impurity was detected in four effervescent tablets (sample 1, sample 2, sample 8, and sample 9) (Table 4). When our results and the PDE value for Hg impurities in supplements were compared, it was seen that there was no risk in the use of 1 effervescent tablet (4.5 g/day). When the HQ values in the samples with Hg elemental impurity are examined in Table 4, it is seen that these values are less than 1 in all samples. Because more than one elemental impurity was not detected, the HI value in each sample was found to be equal to the HQ value. In samples with Hg impurity, the HI value is less than 1. Since the HI values are less than 1, it has been determined that the Hg levels detected in the vitamin C supplements are in the safe range which does not pose a health risk. In the study of Ćwieląg-Drabe *et al*., in 2020, Hg elemental impurities were analyzed in 41 diet supplements. Hg contamination was found in 29.3% of the samples. While some of the contaminations were not at a level to affect human health, the HQ value was greater than one in some of them (27). In our study, 33.3% of the samples had Hg impurities. However, the HQ values in the samples were less than one, indicating that the amount of Hg impurities in our study samples was not at risk of affecting human health.

In Lebanese dietary supplements, Cr, Hg, and Pb elemental impurity analyses were performed in a 2013 study. As a result of the study, it was reported that the Cr, Hg, and Pb levels in the supplements were negligible and did not pose any health risks (45). In our study, the Hg level detected in the supplements is similar to the Hg levels in the literature. It was not found to be at an unsafe level that would pose any health risk. 

Pirsaheb *et al. (*2020) investigated the risk of heavy metal in grains in Iran with the Monte Carlo simulation method (28). At the end of the analysis, it was found that the HQ value of Hg was lower than the allowable limits (28). However, In our study, when the probability calculation of the change in BW, C, and IR values that may affect the HQ value of Hg during supplement consumption is examined, it has been shown that the HQ value of Hg may exceed the hazard level (Figure 2). In order to keep the Hg level, which has a potential risk capacity, at low limits, it is recommended that the necessary risk-reducing measures be taken by the authorities and further studies be carried out.

Chromium (Cr) is a naturally occurring element found in rocks, animals, plants, soil, and volcanic gases. It exists in the environment in two major valences, trivalent Cr {Cr (III)} and hexavalent Cr {Cr(VI)}. Cr (III) compounds are sparingly soluble in water, while Cr (VI) compounds are readily soluble, which can result in higher Cr (VI) levels in water sources. Cr (VI) is more toxic than Cr (III) and is a known carcinogen. Cr (III) is an essential element required for glucose, protein, and fat metabolism. The recommended daily dose of Cr (III) is 50-200 mg. The oral RfD values for Cr (VI) and Cr (III) were 0.003 mg/kg/day (26, 27) and 15000 µg/kg/day (23, 33), respectively. The USEPA estimates that lifetime consumption of these doses or less is unlikely to cause chronic non-cancer effects to occur. In a study by Su *et al.* in 2020, Cr and other elemental impurities were investigated in infant foods. As a sample preparation procedure, a closed system sample preparation procedure was applied by adding HNO_3_ (65%) and H_2_O_2 _(30%) to 0.5 g of infant food. The sample preparation procedure applied in that study is similar to the sample preparation procedure in our study (24). The oral RfD value for Cr was taken as 0.003 mg/kg/day for the risk assessment calculation in the study of Su *et al*. In our study, the oral RfD value was taken as 0.003 mg/kg/day for the calculations, as indicated in Table 3. Cr elemental impurity was detected in three effervescent tablets (sample 5, sample 10, and sample 11) analyzed in our study (Table 4). The reported PDE value of Cr is 11000 µg/day in the literature. For Cr impurities, when our results and PDE values are compared, it is seen that there is no risk in the use of one effervescent tablet (4.5 g/day). When the HQ values of the samples with Cr elemental impurity are examined in Table 4, it is seen that these values are less than 1 in all samples. In each sample, the HI value was found to be equal to the HQ value since more than one elemental impurity was not detected. In the samples with Cr impurities, the HI value is less than 1. Since the risk values are less than 1, it has been determined that the Cr detected in vitamin C supplements does not pose a non-carcinogenic health risk. In a study by Tschinkel *et al.* in 2020, elemental impurities in medicinal plants were analyzed. In this study, a closed-system sample preparation procedure was applied by adding HNO_3_ (65%) and H_2_O_2 _(30%) to 10 gr of medicinal plant. The sample preparation procedure applied in this study is similar to the sample preparation procedure in our study (51). As a result of the analysis of the study of Tschinkel *et al*. in 2020, the Cr content in the plant samples was found to be below the impurity concentration allowed by the USP. In our study, 25% of the samples had Cr elemental impurities. However, the HQ value in the samples was less than one, indicating that the amount of Cr impurity was not at risk of affecting human health.

A 2015 study reported that elemental impurity in medicinal plants and food supplements, Su *et al.* (2020) determined that elemental impurity in infant formulas, and a 2021 study documented that Cr levels in medicinal plants were trace, and the HQ value was less than one (13, 24, 52). The data in our study are in line with the data in the literature. Pirsaheb *et al. (*2020) investigated the risk assessment of heavy metal analysis in grains in Iran with the Monte Carlo simulation method (28). At the end of the analysis, it was found that the HQ value of Cr was lower than the allowable limits. In addition, the CR value of Cr was found to be lower than the allowable limits (28). In our study, when the probability simulation calculation of the change in BW, C, and IR values that may affect the HQ and CR values of Cr during supplement consumption was examined, it was observed that the HQ and CR values of Cr did not exceed the hazard level (Figure 2). Our probability analysis results are similar to those in the literature, showing that the Cr levels in the supplement content are negligible and do not carry non-carcinogenic and carcinogenic risks.

**Table 1:** Analyzed vitamin C supplements and their contents

*Supplement	Formula	Content
S1	Effervescent tablet	L-ascorbic acid (Vit. C), citric acid anhydrate, lactose anhydrate, sodium hydrogen carbonate, polyvinyl pyrrolidone, polyethylene glycol, orange flavoring, sucralose, and riboflavins
S2	Effervescent tablet	L-ascorbic acid (Vit. C), citric acid, sodium hydrogen carbonate, sorbitol, sodium carbonate, inulin, tricalcium phosphate, starch, sucralose, natural orange flavor, beetroot red, and riboflavin 5’ - phosphate sodium
S3	Effervescent tablet	L-ascorbic acid (Vit. C), citric acid anhydrate, sodium hydrogen carbonate, inulin, cyclimic acid and sodium salts, natural lemon flavor, sorbitol, beta carotene, and sodium saccharin
S4	Effervescent tablet	L-ascorbic acid (Vit. C), sodium bicarbonate, citric acid, sorbitol, natural orange juice, corn starch, carotenes, zinc citrate, beetroot red, and sucralose
S5	Effervescent tablet	L-ascorbic acid (Vit. C), citric acid, sodium hydrogen carbonate, natural lemon flavor, sorbitol, beta carotene, and sucralose
S6	Effervescent tablet	L-ascorbic acid (Vit. C), sodium bicarbonate, citric acid anhydrate, sorbitol, natural lemon flavor, corn starch, carotenes, zinc citrate, beetroot red, and sucralose
S7	Effervescent tablet	L-ascorbic acid (Vit. C), citric acid, sodium bicarbonate, cyclimic acid and sodium salts, natural lemon flavor, sorbitol, beta carotene, and sodium saccharin
S8	Effervescent tablet	L-ascorbic acid (Vit. C), citric acid, sodium hydrogen carbonate, sorbitol, tricalcium phosphate, starch, sucralose, natural orange flavor, and beetroot red
S9	Effervescent tablet	L-ascorbic acid (Vit. C), citric acid, lactose anhydrate, sodium hydrogen carbonate, polyvinyl pyrrolidone, polyethylene glycol, natural lemon flavor, and sucralose
S10	Effervescent tablet	L-ascorbic acid (Vit. C), citric acid, lactose anhydrate, sodium hydrogen carbonate, polyvinyl pyrrolidone, polyethylene glycol, natural orange flavor, beta carotene, and sucralose
S11	Effervescent tablet	L-ascorbic acid (Vit. C), citric acid, sodium hydrogen carbonate, sorbitol, starch, sucralose, and natural orange flavor
S12	Effervescent tablet	L-ascorbic acid (Vit. C), sodium bicarbonate, citric acid anhydrate, sorbitol, natural orange flavor, corn starch, carotenes, zinc citrate, beetroot red, and sucralose

* Recommended daily intake (IR) is one effervescent tablet (approximately 4.5 g) per day per capita. Nutritional reference value BRD %; 1250

**Table 2:** Oral Reference dose factor (RfD) and Permitted daily exposure (PDE) limits for elemental impurities of pharmaceuticals

Element	RfD (mg /kg/day)	PDE (µg/day)	Reference
Cd	0.0005	5	32-34
Pb	-*	5	23, 34, 35
As	0.0003	15	33, 34
Hg	0.0003	30	33, 34
Co	0.0003	50	33, 34, 36
V	0.005	100	33, 34
Ni	20000	200	23, 34, 37
Cr	0.003	11000	23, 33, 34, 37, 38
Sb	0.0004	1200	33, 34
Sn	0.6	6000	33, 34

* Not defined RfD for Pb by USEPA. Tolerable weekly intake for Pb is 25 µg/kg (39)

- ^111^Cd; b) ^208^Pb; c) ^75^As; d) ^202^Hg; e) ^59^Co; f) ^51^V; g) ^60^Ni; h) ^52^Cr; i) ^121^Sb; j) ^118^Sn

**Table 3:** Quantification parameters of the inductively coupled plasma-mass spectrometry (ICP-MS) analytical method for elemental impurities

Element	Isotop	LOD (ppb)	Calibration range (ppb)	Calibration curve equation	Calibration curve regression (R^2^) value
Cd	111	0.01	0.1-1000	9628.7863*x + 120.0122	1.0000
Pb	208	0.01	0.1-1000	156222.6825*x + 340.0113	0.9975
As	75	0.01	0.1-1000	23185.9056*x + 360.0078	0.9989
Hg	202	0.001	0.005- 2	9659.9875*x + 200.0025	0.9939
Co	59	0.01	0.1-1000	178431.6850*x + 680.0216	0.9999
V	51	0.002	0.1-1000	164792.2255*x + 3793.9275	0.9994
Ni	60	0.01	0.1-1000	38292.3170*x + 5084.3934	0.9999
Cr	52	0.001	0.01-50	143526.7562*x + 50184.4152	0.9997
Sb	121	0.002	0.01-500	95919.3327*x + 1426.7563	1.0000
Sn	118	0.002	0.01-500	84295.4605*x + 450.0098	0.9995

**Table 4:** Health risk assessment results of elemental impurities in analyzed samples

Sample	ICP-MS level of Elemental impurity (ppb) Mean ± SD	*Calculated amount of elemental impurity (µg/g) Mean ± SD	IR (g)	EDI (mg/kg/day)	HQ	HI	CR
Sample 1 (N:3)							
Cd; Pb; As; Co; V; Ni; Cr; Sb; Sn	< LOD	NC	4.5	NC	NC	NC	NC
Hg	0.005 ± 0.002	0.25 ± 0.015	4.5	1.6.10^-5^	0.054	0.054	NC
Sample 2 (N:3)							
Cd; Pb; As; Co; V; Ni; Cr; Sb; Sn	< LOD	NC	4.5	NC	NC	NC	NV
Hg	0.009 ± 0.001	0.45 ± 0.012	4.5	2.9.10^-5^	0.096	0.096	NC
Sample 3 (N:3)							
Cd; Pb; As; Hg; Co; V; Ni; Cr; Sb; Sn	< LOD	NC	4.5	NC	NC	NC	NC
Sample 4 (N:3)							
Cd; Pb; As; Hg; Co; V; Ni; Cr; Sb; Sn	<LOD	NC	4.5	NC	NC	NC	NC
Sample 5 (N:3)							
Cd; Pb; As; Hg; Co; V; Ni; Sb; Sn	<LOD	NC	4.5	NC	NC	NC	NC
Cr	0.011 ± 0.004	0.55 ± 0.002	4.5	3.5.10^-5^	0.011	0.011	1.767.10^-5^
Sample 6 (N:3)							
Cd; Pb; As; Hg; Co; V; Ni; Cr; Sb; Sn	<LOD	NC	4.5	NC	NC	NC	NC
Sample 7 (N:3)							
Cd; Pb; As; Hg; Co; V; Ni; Cr; Sb; Sn	<LOD	NC	4.5	NC	NC	NC	NC
Sample 8 (N:3)							
Cd; Pb; As; Co; V; Ni; Cr; Sb; Sn	<LOD	NC	4.5	NC	NC	NC	NC
Hg	0.006 ± 0.003	0.30 ± 0.017	4.5	1.9.10^-5^	0.064	0.064	NC
Sample 9 (N:3)							
Cd; Pb; As; Co; V; Ni; Cr; Sb; Sn	<LOD	NC	4.5	NC	NC	NC	NC
Hg	0.008 ± 0.002	0.40 ± 0.013	4.5	2.6.10^-5^	0.086	0.086	NC
Sample 10 (N:3)							
Cd; Pb; As; Hg; Co; V; Ni; Sb; Sn	<LOD	NC	4.5	NC	NC	NC	NC
Cr	0.016 ± 0.007	0.80 ± 0.005	4.5	5.1.10^-5^	0.017	0.017	2.571. 10^-5^
Sample 11 (N:3)							
Cd; Pb; As; Hg; Co; V; Ni; Sb; Sn	<LOD	NC	4.5	NC	NC	NC	NC
Cr	0.013 ±0.003	0.65 ± 0.003	4.5	4.2.10^-5^	0.014	0.014	2.089. 10^-5^
Sample 12 (N:3)							
Cd; Pb; As; Hg; Co; V; Ni; Cr; Sb; Sn	<LOD	NC	4.5	NC	NC	NC	NC

* The calculated amount of elemental impurity; the ppb level values of the effervescent tablets diluted to ppb level in the sample preparation procedure, read in the ICP-MS, were converted to µg/g considering the amount of effervescent tablet used (equation 1)

LOD: below limit of detection, NC: not calculated. N: number of samples

Cr- Hazard quotient (HQ) (a); Cr- Cancer risk (CR) (b); Hg- Hazard quotient (HQ) (c)

## Conclusion

The primary purpose of this study was to evaluate the contamination levels of 10 elemental impurities in 12 vitamin C effervescent tablets and assess the corresponding non-carcinogenic risk. The second purpose was to evaluate the carcinogenic risk of the elements with carcinogenic properties detected in the samples. The non-carcinogenic (HQ and HI) and carcinogenic (CR) health risks were calculated. After that, probabilistic statistics were used. In this study, the analysis of 10 elemental impurities that can be found in the 12 vitamin C effervescent tablets was carried out with ICP‒MS. The metals studied were Cd, Pb, As, Hg, Co, V, Ni, Cr, Sb, and Sn. In the analysis procedure, the closed system microwave sample preparation procedure, which is widely preferred in the literature, was applied. Samples were evaluated over a wide range of calibration standards. In this study, different trademarks of vitamin C were analyzed, and low levels of Hg and Cr were detected in seven samples. Since the HI of the detected elements is less than one, it was determined that they do not pose a risk to human health. The CR value in Cr-containing samples was within the permissible limits. İt was concluded that there is no risk to human health and that there is no critical difference between the supplements, considering the elemental pollutant content among the vitamin C supplements of different trademarks. However, in order to keep the Hg level, which has a potential risk capacity, at low limits, it is recommended that the necessary risk-reducing measures be taken by the authorities and further studies be carried out

## Limitations of study and perspectives for future research

In the study, only vitamin C supplements in effervescent tablet form were analyzed. Among the limitations of the study is that different forms of vitamin C supplements were not analyzed in the study. Also, advanced statistical analyzes could not be performed due to the small number of samples. The data in our study are among the references that can be used for the detection and risk assessment of elemental impurities in pharmaceutical group products. However, for further studies, it is recommended to carry out comprehensive elemental impurity analysis on samples containing different types and different forms of drug groups.

## Authors’ Contributions

FC took part in the analysis of samples, risk assessment, writing of the manuscript and final approval of the version to be published.

## Conflicts of Interest

The authors declare no financial or commercial conflict of interest.
